# Application of Traditional Chinese Medical Herbs in Prevention and Treatment of Respiratory Syncytial Virus

**DOI:** 10.1155/2016/6082729

**Published:** 2016-09-04

**Authors:** Li Li Lin, Jin Jun Shan, Tong Xie, Jian Ya Xu, Cun Si Shen, Liu Qing Di, Jia Bin Chen, Shou Chuan Wang

**Affiliations:** ^1^Jiangsu Key Laboratory of Pediatric Respiratory Disease, Nanjing University of Chinese Medicine, Nanjing 210023, China; ^2^Institute of Pediatrics, The First Clinical Medical College, Nanjing University of Chinese Medicine, Nanjing 210023, China; ^3^Jiangsu Engineering Research Center for Efficient Delivery System of TCM, Nanjing University of Chinese Medicine, Nanjing 210023, China; ^4^Dongzhimen Hospital, Beijing University of Chinese Medicine, Beijing 100700, China

## Abstract

Respiratory syncytial virus (RSV) is a common viral pathogen of the lower respiratory tract, which, in the absence of effective management, causes millions of cases of severe illness per year. Many of these infections develop into fatal pneumonia. In a review of English and Chinese medical literature, recent traditional Chinese medical herb- (TCMH-) based progress in the area of prevention and treatment was identified, and the potential anti-RSV compounds, herbs, and formulas were explored. Traditional Chinese medical herbs have a positive effect on inhibiting viral attachment, inhibiting viral internalization, syncytial formation, alleviation of airway inflammation, and stimulation of interferon secretion and immune system; however, the anti-RSV mechanisms of TCMHs are complicated, which should be further investigated.

## 1. Introduction

Respiratory syncytial virus (RSV) is classified as a nonsegmented, negative-sense, membrane-bound RNA virus of the family Paramyxoviridae, spread by droplets, and causing repeated airway infections [1]. Respiratory syncytial virus is the most common cause of hospitalization in infants and children with severe acute lower respiratory infections. Additionally, it is the third most common cause of death in children, through the development of fatal pneumonia, after pneumococcal pneumonia and* Haemophilus influenzae* type b infection. It had been estimated that between 66 000 and 199 000 children younger than 5 years died of RSV-associated diseases in 2005, with 99% of these deaths occurring in developing countries [2]. In the 1960s a formalin-inactivated vaccine (FI-RSV) given intramuscularly did not reduce the frequency of infection and among infected children (particularly in the youngest age cohort, immunized between 2 and 7 months of age) 80 percent required hospitalization and 2 died [3–5].

Due to the nature of the virus and the mode of infection, reinfections are a common event, suggesting that naturally acquired immunity does not provide long-lasting protection. This has made the development of an effective vaccine impossible as yet [6]. Currently, the Food and Drug Administration (FDA) has approved prophylactic drugs for RSV, including palivizumab and ribavirin, administered along with symptomatic treatment and supportive care [7]. While palivizumab, a humanized monoclonal antibody directed against the F protein RSV, effectively prevents RSV infection, it is expensive and ineffective in the management of an established RSV infection [8]. Ribavirin has been shown to exhibit potent activity against RSV in vivo and in vitro; however comparisons of the results of both animal and cell testing in humans have not yet been completed [9–11]. Furthermore, ribavirin is limited due to the side effects [11, 12]. There are any antiviral agents against RSV that are currently under development.

This review aims to explore evidence available on preventative measures against RSV infection, discuss the current status of anti-RSV research on TCMH-derived active compounds, TCMHs, and traditional Chinese medical formulas (TCMFs), and finally highlight the challenges, issues, and future directions of using TCMHs in clinical practice.

## 2. Evidence Supporting the Efficacy of TCMHs

Traditional Chinese medical herbs are the most important components of the traditional Chinese medicine (TCM) system, which have been reported to cure infectious diseases, in the form of hot water extracts, for almost 2,000 years. Over 10,000 herbal medicines and 100,000 recipes have been documented in ancient literature [13].

Nowadays, TCMHs are widely used for the prevention and treatment of viral infectious diseases in China and many other Asian countries. They are associated with advantages such as lower toxicity, economical and multifunctional use in regulating immunological function, and viral destruction [14]. This reduction in viral load allows for the screening of effective medications. However, the international community remains uncertain about the efficacy of TCMHs, due to the lack of supporting clinical evidence collected under international standards (randomized, placebo-controlled, double-blind, and multicentered clinical studies). Governments have put forward support aimed at the international regulatory approval of TCMHs. Leading the pack is compound T89 (also known as Dantonic, a traditional Chinese medical product by Tasly Pharmaceuticals, China), which is used in China for the management of ischemic heart disease. Dantonic may become the first traditional Chinese medicine to receive Food and Drug Administration (FDA) approval in the United States [15]. Moreover, the Yinhuang granules (tablet) and Xiasangju granules have been approved by the China Food and Drug Administration (SFDA) for the treatment of RSV-infectious diseases [15]. The 2015 Nobel Prize in Medicine was awarded to Youyou Tu for the discovery of artemisinin, a drug isolated from a plant, which has significantly reduced the mortality rates for patients with malaria. This acknowledgement has increased the optimism of the natural product community worldwide [16].

## 3. Prevention

Prevention is the most important aspect of healthcare, and because of the nature of the respiratory syncytial virus and mode of infection, reinfections are a common event [6]. Live RSV can survive on surfaces for several hours; moreover, direct or indirect contact with the nasopharyngeal secretions or droplets (sneezing, coughing, and kissing), fomites, and food from infected patients can potentially transmit RSV [7]. Therefore, without effective preventive measures, the mortality and economic burden of the disease will increase.

### 3.1. RSV F Protein Neutralizing Antibodies

The respiratory syncytial virus depends on the host cell to complete its replication cycle, which includes attachment and entry into the host cell, transcription of viral mRNA, viral genome replication, protein synthesis, and the assembly and budding of progeny virus particles [15]. The F protein of RSV accumulates in the host membrane and surrounds the budding progeny viruses, thus spreading the infection to adjacent cells and exacerbating the infection. The F protein is therefore considered a potential candidate for the development of possible preventive measures. Palivizumab is a humanized monoclonal antibody (IgG) directed against an epitope in the A antigenic site of the F protein of RSV. Palivizumab-N, a plant-based F neutralizing antibody, was experimented in phages and plants. Successful palivizumab-N production was observed in the* Nicotiana benthamiana* plant system, which offered glycosylation and high production at low upstream and equivalent downstream cost, when compared to mammalian derived palivizumab [7].

### 3.2. Boost Resistance against RSV by Modulating Immune Response

Following coevolution with the host, many viruses have established sophisticated mechanisms to interact with the host immune system for immune evasion. Among the classes of antiviral agents, immunomodulators are the most abundant in TCMHs [15]. Traditional Chinese medical herbs have been shown to enhance antibody production, T cell proliferation, the expression of antigen specific CD4+ and CD8+ responses, and increase of the titers of IgG1, IgG2a, and IgA, along with the function to balance Th1, Th2/Th17 type immune response [17]. Interferon- (IFN-) *γ* and interleukin- (IL-) 12 are representatives of Th1 type cytokines; interleukin 4 and interleukin 5 belong to Th2 type cytokines, and IL-6 and IL-17a are representatives of Th17 type cytokines, all of which play key roles in Th2/Th17 responses [18, 19]. And, recently, macrophages have been identified as stimulation cells by interaction with Th2 cytokine IL-4 [20]. Traditional Chinese medical herbs were reported to simultaneously promote Th1-response, including increased levels of Th1 type cytokine secretion and activation of alveolar macrophages, suppress Th2/Th17-responses, and maintain the balance between Th1 and Th2/Th17 cells for the prevention and treatment of diseases.

Moreover, TCMHs have been previously reported to offer efficient protection against RSV and significantly decrease viral load and inflammation [15]. It is well-known that the production of inflammatory mediators is regulated by a transcription factor, NF-*κ*B, which plays a pivotal role in inflammation due to its ability to induce the transcription of inflammatory genes [21–23]. Toll-like receptor 4 (LR4) is also a factor expressed on the respiratory epithelium and macrophages and appears to play a central role in mediating both the antiviral and inflammatory responses of the innate immunity to combat viral infections [22]. Along with direct antiviral activity, TCMHs could be beneficial in preventing RSV infection due to its immune-modulatory effect, producing of IFN-*β* and TNF-*α*. Respiratory syncytial viral infection can induce cellular production of IFN-*β* and TNF-*α*. Both IFN and TNF contribute to the innate immunity against viral infection [24] and TNF triggers multiple antiviral mechanisms and synergizes with IFN in promoting antiviral activities [25].

Resveratrol, a compound synthesized by at least 72 plant species [26], exhibited certain abilities in the inhibition of RSV replication, inflammation, the level of IL6, virus-induced TIR-domain-containing adapter inducing interferon-*β* (TRIF), and TANK binding kinase 1 (TBK1) protein expression [27]. Moreover, it has been shown to prevent airway inflammation/hyperresponsiveness during RSV infection in mice, suggesting its applicability in reducing RSV-induced airway symptoms. Additionally, it has also been shown to reduce IFN-*γ* levels associated with RSV-mediated airway inflammation [28].

In Table 1, we have provided evidences of medicinal herbs used in TCM for the prevention of RSV. This novel approach seems to be a promising and safe prevention option against RSV.

## 4. Treatment

### 4.1. TCMH-Derived Active Compounds

Drug use from natural products has progressed to the isolation of active compounds. Natural products and particularly medicinal plants remain an important source of new drugs, new drug leads, and new chemical entities [58, 59]. Drug discovery from nature led to the isolation of early drugs such as quinine, artesunate, and arsenic trioxide, which are active ingredients effectively extracted from natural products and have since proven to be clinically successful. Quinine is made from cinchona bark and artesunate is derived from* Artemisia annua*, and both are used for the treatment of severe malaria [60, 61]. Arsenic trioxide, extracted from white arsenic, is used for the treatment of acute promyelocytic leukemia [62, 63].

Traditional Chinese medical herbs have been utilized as anti-infectious medicines for thousands of years in China and offer various anti-infectious compounds, particularly for antiviral. An increasing number of TCMH-derived active compounds with antiviral activity are garnering evidence of experimental efficacy. As shown in Green Bae's research, (−)-(R)-nyasol, (−)-(R)-4′-O-methylnyasol, and broussonin A, three structurally known compounds isolated from the rhizomes of* A. asphodeloides*, exhibited potent antiviral activities against the RSV-A2 strain propagated in HEp-2 cells. The results showed IC50 values of 0.85, 0.39, and 0.62 *μ*M, respectively, slightly more potent than that of the positive control ribavirin (IC50 = 1.15 *μ*M) [64].  Table 2 details a list of anti-RSV active compounds derived from TCMHs verified by experiments in vivo or in vitro.

#### 4.1.1. The Characteristics of Active Compounds in TCMHs

A vast amount of basic research has been conducted on TCMHs. Therefore, the isolation and characterization of pharmacologically active compounds from TCMHs and the testing of their pharmacological activities, in the pursuit of new drug discovery, are now necessary [65]. When compared to random screening from a combinatorial chemical library, TCMHs are drugs with a long history of medical use and could be used as the markers for selecting active ingredients from herbal medicines. It would therefore be more efficient to screen active compounds from TCMHs. Furthermore, in biological screening assays, TCMH-derived active compounds often have better pharmacokinetics and bioavailability [15].

Previous studies, as shown in Table 2, have provided evidence of the direct anti-RSV activity of compounds derived from TCMHs. Notwithstanding the failure to discover new chemical structures during drug discovery from medicinal plants, known compounds with new biological activities, such as the inhibition of RSV replication and activation of the immune system, which can provide important drug leads, may be found [66]. Moreover, for countries with limited resources, TCMHs-derived active compounds will serve as a gateway for the merging of modern drug discovery.

However, even with these unique advantages, screening programs focused on identifying potential anti-RSV agents from TCMHs are using conventional methods. In order to compete with other drug discovery methods, efforts are ongoing to replace these traditional methodologies with modern sample preparation and extraction procedures. Therefore, TCMH research needs to continually improve the speed of screening, isolation, and structure elucidation processes. The most challenging issues are understanding the operative mechanisms to identify TCMH-derived active components, as each kind of TCMH contains multiple active components with multiple targets. Some of these components work directly on the therapeutic targets, whereas others may enhance the bioavailability [59]. Therefore, more efforts are needed in the research of compounds in TCMH assays.

It is known that cell-based assays are often initially carried out for the evaluation of whole extracts that show clinical evidence of antiviral activity. Without larger randomized, double-blind, placebo-controlled multicenter clinical trials, approval of TCMH-derived active compounds will be difficult through international regulatory agencies such as the Food and Drug Administration (FDA) of the United States of America or other equivalent European counterparts.

Above all and in long term, TCMHs still offer a great prospect in the field of pharmaceutical developments. The characteristics of TCMHs offer opportunities for finding active and novel chemical structures against a variety of therapeutic targets.

### 4.2. Traditional Chinese Medical Herbs (TCMHs)

Traditional Chinese medicine theory is in line with seasons, circumstances, and individuality. For example, in summer, “*Agastache rugosa*” or “*Eupatorium*” is commonly used in northern China (colder district) to ease the summer-heat and “*ephedra*” and to relieve exterior syndrome (“exterior syndrome” refers to lesions with mild conditions. The symptoms are aversion to cold, fever, headache, body pain, stuffy nose, no sweat, and so on) by diaphoresis while in southern China (warmer district), “honey-fired* ephedra*” is frequently used because of its mild function of stimulating the secretion of sweat. Moreover, the TCM treatment should vary between individuals. For the children with upper respiratory tract infection, the treatment method aims to clear the exterior evil (“evil” is the general name of all kinds of pathogenic factors; “exterior syndrome” discussed above is caused by exterior evil), while, in the elderly, the treatment method aims to strengthen body resistance and relieve the exterior syndrome.

In both 2001 and 2002, approximately one-quarter of the bestselling drugs worldwide were natural products or were derived from natural products [59]. Recent publication shows that TCMHs account for 10% of the prescription drugs in China. They are perceived as harmless and natural and are widely used in several parts of the world individually or in combination [15].

#### 4.2.1. The Characteristics of TCMHs

Traditional Chinese medical herbs are the most important components of the TCM system, which have long been used for the multiple combinations of compounds in the form of processed natural products. Experimental findings of TCMHs with the identification of potent antiviral activities against RSV should be made available to the healthcare providers practicing traditional medicine. From a clinical perspective, diseases are complex and variable, combined with complex clinical symptoms. Traditional Chinese medical herbs contain multiple active components with multiple targets, which may be a better choice to achieve multiacting treatment.

Traditional Chinese medicine theories originate from the profound experiences of practitioners with understanding of ancient Chinese medicine, and the methods of application have been passed down through oral history. It is suggested that hot water extracted TCMHs would exhibit direct antiviral activity. In this review, we summarized experiments in vivo or in vitro (Table 3) that used aqueous extracts of TCMHs for treating RSV-infectious diseases. The objective of these experiments was to review the potential uses of natural products for the treatment of infections caused by RSV.

Ma et al. recently tested 44 medicinal herbs, used in the TCM system, for treating virus-infectious diseases. The medicinal herbs were tested for antiviral activities against RSV using a cytopathologic effect (CPE) assay. Twenty-seven of the 44 medicinal herbs showed moderate or potent antiviral activities against RSV, with a 50% inhibition concentration (IC50) ranging from 6.3 to 52.1 g/mL and a selectivity index (SI) ranging from 2.0 to 32.1. The control group, ribavirin, had an SI of 24 [44].

This evidence illustrates that natural products can be a rich source for the discovery of potent anti-RSV agents.

#### 4.2.2. The Bottleneck Problems of TCMHs

Although traditional natural products have played an important role in drug discovery, in the past few years, many pharmaceutical companies have scaled down or terminated their natural product research [59]. Drug discovery programs already follow a challenging process: the design, determination, and implementation of appropriate, clinically relevant, high-throughput bioassays of drugs, and TCMH discovery programs are more complicated and lengthier than other drug discovery methods.

Despite the recognized therapeutic benefits and the expanding use of medicinal plants worldwide, TCMHs still lack robust evidence from evidence-based medicine (EBM) research, especially the supporting clinical evidence collected under international standards (randomized, placebo-controlled, double-blind, and multicentered clinical studies). As a result, it is a challenge for TCMHs to be accepted by the Western medicine community and mainstream healthcare systems.

Though much debate remains on the feasibility and the validity of applying the EBM criteria to this traditional practice, for the integration of TCM into the Western healthcare system, it is essential to demonstrate its efficacy and safety through high level evidence in accordance with EBM procedures.

### 4.3. Traditional Chinese Medicine Formulas (TCMFs)

Based on TCM theory, a remedy often has a principle of hierarchy, the so-called “monarch,” “minister,” “assistant,” and “guide components” [53], and the functions differ among the different roles. The basic principle of TCM treatment is to keep the balance between Yin and Yang (two opposite as well as relevant agents, the main components of the universe in Chinese philosophy) and Qi and blood (two basic components of the human body, with the functions of maintaining the life).

A TCM formula contains multiple active herbs with multiple compounds and targets. Some of these components (“monarch” and “minister”) work directly on the therapeutic targets, while others (“assistant” and “guide components”) enhance the bioavailability or counteract drug toxicity of the medicine [15]. This review summarized some TCMFs and Chinese patent medicines in Tables 4 and 5, which had anti-RSV activities, supporting their possible use in managing RSV infection.

#### 4.3.1. The Characteristics of TCMFs and Chinese Patent Medicines

Traditional Chinese Medicine Formulas have been used as remedies against infectious diseases for thousands of years due to their significant anti-inflammatory, antimicrobial, and antiviral activities, with little or no adverse effects [44, 67]. Therapeutic effects of mixed formulas are based on the synergic action of its mass constituents [67]. Currently, Chinese patent medicines, with convenient dosage forms, represent the majority of drugs on the market. Anti-RSV Chinese patent medicines (during infection) are usually considered “cold” herbs and are applied to remove the heat-evil (“heat evil” related to the clinical manifestations characterized by ulcers local swelling and heat pain), expel superficial evils (superficial evils lead to “exterior syndrome,” which is explained in previous 17th paragraph), and dispel toxins from the body, such as Shuang Huang Lian oral liquid, Jin Xin oral liquid, Qing Fei oral liquid, or Xiasangju granules (Table 4). The Xia Sang Ju granule was approved by the China Food and Drug Administration (SFDA), and Jin Xin oral liquid, a traditional Chinese patent medicine, has been widely used for decades to treat virus-infectious pneumonia in the Jiangsu Province Hospital of Nanjing University of Traditional Chinese Medicine, Nanjing, China. Following recovery from an infection, Bu Shen Yi Qi formula is administered, which is a traditional Chinese herbal formula composed of herbs used to tonify Qi of the kidney, that is, the strengthening of the body's resistance to eliminate pathogenic factors. The anti-RSV mechanism of Bu Shen Yi Qi formula was investigated in the Th1, Th2, and Th17 cells [50].

Based on the improvements in experiments in vivo or in vitro, a number of Chinese patent medicines are currently undergoing FDA clinical trials in the hope of fulfilling EBM research criteria. This includes Danshen dripping pill (*Salvia miltiorrhiza*,* Radix notoginseng*, and Borneol) for stable angina in a phase III trial, Fu zheng Hua yu capsule (*Salvia miltiorrhiza*, peach seed, pine pollen,* Gynostemma pentaphylla*, and* Schisandra chinensis*) for liver fibrosis, Kang lai te injection (coix seed oil and excipients) for cancer, and the Xue zhi kang capsule (red yeast) for hyperlipidemia in phase II trials [71].

Traditional Chinese Medicine Formulas rely on written traditional Chinese medicine and an educational system of TCM scholars. According to Shanghan Lun (Treatise on Cold Pathogenic Diseases), written by Zhang Zhongjing, TCMFs should be administered three times. A dosage three times a day is recommended to avoid an overdose and to ensure the better absorption of drugs. This is to ensure a balanced blood drug concentration, according to modern medicine [72].

In TCM theory, the quality of the decoction is the key to cure diseases successfully; hence detailed requirements of the decocting methods should be illustrated. The methods of decocting medicinal herbs were passed down through the form of hot water extracts [44]; the oral form of the decoction would exhibit major effects, such as direct antiviral activity. The decocting time differs from herbs to herbs. For aromatic plants, such as* Mentha* and* Agastache rugosa*, the drug efficacy will be lost with lengthened decocting; therefore these plants should be decocted for only 5–10 minutes, while for plants with a certain level of toxicity, such as* Aconitum carmichaelii *and* Pinellia ternata*, decocting should be at least 1 hour to reduce its toxicity.

Compared to Western medicines, advantages of TCMFs include individual treatment based on “multicomponents, multichannels, and multitargets” application and syndrome differentiation. The characteristics of TCMFs are to balance the body and improve clinical symptoms of a patient. Considering these unique characteristics, development of TCMFs, in Tables 4 and 5, with anti-RSV activity is of great importance. However, this clinical application needs further clinical validation.

#### 4.3.2. The Bottleneck Problems of TCM Formulas

With the development of science and technology and the increasing need of modern clinical practice, the disadvantages of oral TCM preparations have gradually been realized. These include single administration, old formulation, large dosage, slow effect, and outdated preparation and transport methods.

The development of Chinese patent medicines has aided the classification; however, in treating acute and severe diseases, TCM formulas are criticized for their slow effect and large dosage. This prevents TCM from being able to solve urgent medical diagnoses. Moreover, dosage needs to be improved: for a convenient use of the drug and the improvement of the therapeutic effect, the dosage needs to be improved while upholding the active effect. More experiments and clinical trials are needed in the field of TCMFs.

## 5. Future Directions in the Prevention and Treatment of RSV

### 5.1. Attachment/Internalization Inhibitors

The spread of RSV is initiated through viral attachment and internalization. The severity of the RSV-infectious disease is positively correlated with the viral load [44]; therefore, inhibition of viral spread between airway mucosal cells to minimize viral replication and the decrease viral load is essential for disease control.

Traditional Chinese medical herbs can prevent the respiratory viral infection induced by RSV, by inhibiting viral attachment and penetration and reducing the increased susceptibility of the cell to the invasion of RSV. From details in Table 2 to Table 5, conclusions can be made that TCMHs can effectively inhibit viral attachment and cell internalization, in order to minimize the viral spread and replication.

### 5.2. Enhancement of Immunity

Traditional Chinese medical herbs can counteract RSV infection not only through the direct inhibition of viral attachment, internalization, or replication, but also by the enhancement of immunity (Table 6). They are considered to be less toxic and may warrant further evaluation as a possible agent for RSV prevention.

Antiviral cytokines play an important role in the immune system process of preventing and inhibiting RSV infection. Traditional Chinese medical herbs can stimulate antiviral cytokines such as IFN-*β* and TNF-*α*, before and after RSV inoculation. TCMHs have also been shown to keep the balance between Th1 and Th2/Th17 immune response, enhance antibody production and T cell proliferation, enhance the expression of antigen specific CD4+ and CD8+ responses, and increase the titers of IgG1, IgG2a, and IgA [17].

### 5.3. Future Directions

Despite the evident successes of drug discovery from medicinal plants, future endeavors face many challenges. Efforts are needed to continuously improve the quality and quantity of TCMH-derived compounds, herbs, and formulas that enter the drug development phase, in an effort to compete with other drug discovery efforts [59]. The future of current TCMH research is to meet international standards, including evidence-based efficacy (particularly through multicenter, randomized, double-blind, placebo-controlled clinical trials), safety assessment, and quality and quantity control. The aims for the future are that large TCM-focused databases become available and innovative strategies are applied to improve the process of plant collection. Moreover, it is necessary to establish a standardized and centralized research system, aimed at achieving a better understanding of medicinal chemistry and the mechanism of action of TCHMs. The challenges associated with anti-RSV herbs are many. However, at the current pace of scientific research and development, traditional Chinese medical herbs can play a role in effective prevention and treatment of RSV-infectious diseases.

## 6. Conclusions

Prevention is the most important aspect of healthcare; however due to the nature of the RSV and its mode of infection, a safe and effective RSV vaccine has remained elusive. Traditional Chinese medical herbs can enhance immunity to prevent RSV infection, and, furthermore, TCMHs can form part of anti-RSV medications by inhibiting viral attachment, viral penetration, and reducing the increased susceptibility of the cell to the invasion of RSV. This review details evidence that TCMH can be effective as preventative or treatment medications for RSV, the advantages of lower cost, better patient outcomes, and fewer adverse reactions. From the research that has been conducted in this review, we can see great potential value of conducting trials using TCMH. However, further research, especially focused on the toxicity issues, is required to develop the level of evidence to support TCMH to be applied to practice.

## Figures and Tables

**Table 1:** Aqueous extracts of TCMHs in prevention of RSV.

Herbs	Used part	Prevention activity	References
*Fresh ginger*	Rhizome	Showed its better effect when given before viral infection: viral attachment ↓; viral internalization ↓	[29]
		In HEp-2 cells: IC50 was 212.7 *μ*g/mL (2 h before); in A549 cells: IC50 was 26 *μ*g/mL (2 h before) IC50 was 82.8 *μ*g/mL (1 h before)	
		Secrete IFN-*β* that contributed to the inhibition of viral replication	

*Ginseng* (naturally dried)	Root	Anti-inflammatory functions: less weight loss; diminishing pulmonary inflammatory response	[30]
		Inducing host immune responses toward Th1 type immunity: IgG2a isotype antibodies ↑; IFN-*γ* ↑; IL-4 ↓; CD4 T cell infiltration ↓: cell numbers of CD4+ T (CD3+ CD8−) cells ↓; the ratio of CD8+/CD4+ T cells ↑	

*Panax Korean red ginseng* (fermented red)	Root	In the prevention of RSV infections: improved survival of human lung epithelial cells against RSV infection; inhibited RSV-induced cellular oxidative damage	[31]
		Play a role in priming the host immune system: enhanced IFN-*γ* production following RSV viral infection; blocked induction of RSV-induced proinflammatory gene expression in the human alveolar epithelial cell line	

*Radix Glycyrrhizae*	Rhizome	It is more effective when given before viral inoculation (*P* < 0.0001): inhibition of viral attachment (*P* < 0.0001) and penetration (*P* < 0.0001)	[32]
		In HEp-2 cells: IC50 was 78.0 *μ*g/mL (2 h before) IC50 was 99.7 *μ*g/mL (1 h before); in A549 cells: IC50 was 18.8 *μ*g/mL (2 h before) IC50 was 33.8 *μ*g/mL (1 h before)	
		Stimulate mucosal cells to secrete IFN-*β* to counteract viral infection	

IL-4: interleukin-4; IFN-*γ*: interferon-*γ*; IFN-*β*: interferon *β*; Th1 type immunity: T helper type 1 (Th1) immune; A549: human lung carcinoma cell; HEp-2: human larynx epidermoid carcinoma cell.

**(a):** 

Herbs	Compounds	Anti-RSV activity	References
*Folium Isatidis*	Fraction III; fraction IV	RSV replication ↓ (SI: 13.06; >24.33; MTT: 11.67; >22.32) The ribavirin group (SI: <28.04; MTT: 40.52)	[33]

*Flos Pueraria Omeiensis*	Genistein, tectorigenin	Possess potent antiviral activity against RSV (CC50: 450; 500 *μ*g/mL; IC50: 12.5; 30 *μ*g/mL, SI: 36; 16.7); the ribavirin group: (CC50: 62.5 *μ*g/mL; IC50: 3.0 *μ*g/mL; SI: 20.8)	[34]

*Radix Gentianae*	RG2-1 [35] RG3-1 [36]	RSV replication ↓ (TC50: 10.66; 5.29 mg/mL; EC50: 420; 262.95 *μ*g/mL; TI: 25.38; 20.12) [35, 36]; the ribavirin group (TC50: 1.18; 1.15 mg/mL; EC50: 30.88; 15.48 *μ*g/mL; TI: 38.21; 74.92) [35, 36]	[35, 36]

*Herba Patriniae*	AP3 [37] AP4 [38]	RSV replication ↓; RSV entry ↓ (TC50: 11.45; 10.89 mg/mL; EC50: 0.0986; 0.0801 mg/mL; TI: 116.12; 135.95) [37, 38]; the ribavirin group (TC50: 0.076, 1.97 mg/mL; EC50: 0.00143, 0.036 mg/mL; TI: 53.45, 54.72) [37, 38]	[37, 38]

*Litchi Chinensis*	Flavonoids	RSV replication ↓ (TC50: 152.9 mg/mL; IC50: 58.6 mg/mL; TI: 2.6); the ribavirin group (TC50: 154.9 mg/mL; IC50: 57.1 mg/mL; TI: 2.7)	[39]

*Radix Glycyrrhizae*	18*β*-GA [32] GD4 [40]	RSV replication ↓ (IC50: 4.3–4.5 *μ*g/mL; CC50: 71.5–76.3 *μ*g/mL; SI: 15.9–17.7) [32]; (EC50: 28.73 *μ*g/mL; TI: 8.0) [40]; the ribavirin group (EC50: 5.67 *μ*g/mL, TI: 215.0) [40]	[32, 40]

*Ligustrum lucidum*	Oleuropein	Significant antiviral activities against RSV (TC50: 562.5 *μ*g/mL; IC50: 11.7 *μ*g/mL; TI: 48); the ribavirin group (TC50: 62.5 *μ*g/mL; IC50: 2.6 *μ*g/mL; TI: 24)	[41]

*Lophatherum gracile*	Flavone 6-C-monoglycosides	Potent antiviral activities against RSV (CC50: ranging from 254.5 ± 6.4 to 362.6 ± 15.4 *μ*g/mL; IC50 ranging from 5.7 to 50 *μ*g/mL, SI ranging from 5.6 to 63.6 *μ*g/mL); the ribavirin group (CC50: 62.5 ± 3.2 *μ*g/mL; IC50: 3.0 ± 0.4 *μ*g/mL; SI: 20.8)	[42]

*Pithecellobium clypearia*	Quercetin	Possess potent antiviral activity against RSV (IC50: 2.5 *μ*g/mL; SI: 180); the ribavirin group (IC50: 3.0 *μ*g/mL; SI: 20.8)	[43]

*Sophora flavescens*	Anagyrine, oxymatrine, sophoranol	Potent antiviral activities against RSV (IC50: 10.4; 10.4; 10.4 *μ*g/mL; CC50: 250; 125; 250 *μ*g/mL; SI: 24.0; 12; 24); the ribavirin group (IC50: 2.6 *μ*g/mL; CC50: 62.5 *μ*g/mL; SI: 24)	[44]

TC50 is 50% of toxic concentration; EC50 is the concentration for 50% of maximal effect; IC50 is the concentration of the sample required to inhibit virus-induced CPE 50%; CC50 is the concentration of the 50% cytotoxic effect; selective index (SI) or therapeutic index (TI): CC50/IC50 or TC50/EC50 or TC50/IC50; MTT assay is a colorimetric assay for assessing cell metabolic activity.

**(b):** 

Herbs	Compounds	Anti-RSV activity	References
*Scutellaria baicalensis*	Wgonin oroxylin A	Potent antiviral activities against RSV (IC50: 7.4; 14.5 *μ*g/mL; CC50: 119.2; 58.1 *μ*g/mL; SI: 16.1; 4.0); the ribavirin group (IC50: 2.6 *μ*g/mL; CC50: 62.5 *μ*g/mL; SI: 24)	[44]

*Selaginella uncinata*	Uncinoside A; uncinoside B	Potently inhibit RSV infection (IC50: 6.9; 1.3 *μ*g/mL; TC50: 82.5; 83.3 *μ*g/mL; TI: 12; 64); the ribavirin group: (IC50: 2.6 *μ*g/mL; CC50: 62.5 *μ*g/mL; TI: 24)	[45]

*Wikstroemia indica*	Daphnoretin	RSV replication ↓ (IC50: 5.87 *μ*g/mL; SI: 28.17); the ribavirin group (IC50: 3.05 *μ*g/mL; SI: 21.4)	[46]

*Radix wikstroemiae*	Genkwanol B; genkwanol C; stelleranol	Potent antiviral activities against RSV (IC50: 9.6; 6.6; 10.2 *μ*g/mL; CC50: 106.1; 145.3; 161.5 *μ*g/mL; SI: 11; 21.9; 15.8); the ribavirin group (IC50: 11.9 *μ*g/mL; CC50: 256.1 *μ*g/mL; SI: 21.6)	[47]

*Youngia japonica*	Fractions 10 [26]; fractions 11 [26]; 3,4-dicaffeoylquinic acid [48]; 3,5-dicaffeoylquinic acid [48]	Syncytium formation of RSV ↓; potent antiviral activities against RSV (IC50: 3.0–6.0 *μ*g/mL; MNCC: 200 *μ*g/mL) [26]; (IC50: 0.5 *μ*g/mL) [48]; the ribavirin group (IC50: 3.0 *μ*g/mL, MNCC: 31 *μ*g/mL) [26]; the ribavirin group (IC50: 2.5 *μ*g/mL) [48]	[26, 48]

TC50 is 50% of toxic concentration; EC50 is the concentration for 50% of maximal effect; IC50 is the concentration of the sample required to inhibit virus-induced CPE 50%; CC50 is the concentration of the 50% cytotoxic effect; selective index (SI) or therapeutic index (TI): CC50/IC50 or TC50/EC50 or TC50/IC50; MNCC: the maximal concentration of the sample that did not exert toxic effect detected by microscopic monitoring after 72 h of incubation.

**Table 3:** Aqueous extracts of TCMHs inhibiting RSV.

Herbs	Used part	Anti-RSV activity			References
		IC50 (*μ*g/mL)	CC50 (*μ*g/mL)	SI	
*Bupleurum chinense*	Root	36.8	441.3	12.0	[44]
*Gardenia jasminoides*		21.0	112.1	5.3	
*Isatis indigotica*		50.0	1000	20.0	
*Ipomoea cairica*		52.1	833.3	16.0	
*Polygonum multiflorum*		52.1	>1000.0	>19.2	
*Platycodon grandiflorum*		<44.1	352.5	>8.0	
*Polygonum cuspidatum Sieb.*		<13.0	>200.0	>15.4	
*Scutellaria baicalensis*		<10.4	>62.5	6.0	
*Sophora flavescens*		52.1	833.3	16.0	
*Tinospora capillipes*		17.2	275.8	16.0	
*Paeonia suffruticosa*	Root cortex	26.0	>833.3	>32.0	
*Phragmites communis*	Rhizome	46.5	185.8	4.0	
*Andrographis paniculata*	Aerial parts	27.6	295.0	10.7	
*Artemisia capillaries*		13.0	208.3	16.0	
*Schizonepeta tenuifolia*		49.2	196.9	>4.0	
*Scutellaria indica*		31.3	350.0	11.2	
*Perilla frutescens*	Leaf	37.6	255.6	6.0	
*Lonicera japonica*	Flower bud	50.0	>1000.0	>20.0	
*Dendranthema morifolium*	Flower	50.5	269.2	5.3	
*Forsythia suspense*	Fruit	50.0	1000.0	20.0	
*Prunella vulgaris L.*	Fruit spike	<10.4	>62.5	6.0	
*Callicarpa nudiflora*	Whole plant	37.5	800.0	21.3	
*Patrinia villosa*		<13.0	416.7	>32.0	
*Sarcandra glabra*		50.0	1000.0	20.0	
*Selaginella sinensis*		50.0	>62.5	>20.0	
*Blumea laciniata Callicarpa*		15.6	243.8	15.5	[49]
*Laggera pterodonta*		15.6	500.0	31.8	
*Mussaenda pubescens*		512.5	32.0	16.0	
*Schefflera octophylla*	Leaf stalk	12.5	500.0	40.0	
Ribavirin [44]	—	2.6	62.5	24.0	[44]
Ribavirin [49]	—	3.0	62.5	20.8	[49]

IC50 is the concentration of the sample required to inhibit virus-induced CPE 50%; CC50 is the concentration of the 50% cytotoxic effect; selective index (SI) or therapeutic index (TI): CC50/IC50.

**(a):** 

Names	Composition			Mechanisms and results	References
	Plant or mineral	Weight (g) (%, w/w)	Used part		
Bu Shen Yi Qi Tang (BSYQF) (granules)	*Radix Astragali*	46.15%	Root	Airway inflammation ↓; RSV replication ↓; F protein expression of RSV ↓ (*P* < 0.05)	[50]
	*Herba Epimedii;* *Rehmanniae Radix*	30.77% 23.08%	Aerial part, root tuber	Regulating the balance between Th1 and Th2/Th17 responses: Th1-response ↑: CD4+ T cell proportion ↑; T-Bet ↑; macrophages ↑; IFN-*γ* ↑; IL-12 ↑ Th2/Th17 cell proportions ↓: GATA3 ↓; STAT6 ↓; ROR*γ*T ↓; IL-4 ↓; IL-5 ↓; IL-6 ↓; IL-17a ↓	

Shuang Huang Lian oral liquid (SHL)	*Scutellariae baicalensis Georgi*	25.00%	Rootstalk	RSV replication ↓; the virus titer ↓; airway inflammation ↓; IL- 8 ↓; the SHL group: TC50 > 160 *μ*g/mL; the ribavirin group was similar to the SHL group (*P* > 0.05); no adverse reaction was found	[51, 52]
	*Lonicera japonica*	25.00%	Flower bud		
	*Forsythia suspensa*	50.00%	Fruit		

Sheng Ma Ge Gen Tang (SMGGT)	*Pueraria lobata Ohwi*	33.33%	Radix	The crude extract of SMGGT was more effective when given before viral infection (*P* < 0.0001): viral attachment ↓; RSV internalization ↓	[24]
	*Paeonia lactiflora Pallas *	22.22%	Radix		
	*Cimicifuga foetida L. *	22.22%	Rhizoma	The SMGGT group: CC50: 3000 *μ*g/mL; IC50: 34.2–82.8 *μ*g/mL; the ribavirin group was similar to the SMGGT group (*P* > 0.05)	
	*Glycyrrhiza uralensis Fischer et DC *	11.11%	Radix		
	*Zingiber officinale Roscoe *	11.11%	Root-like stem	Epithelial cells secrete IFN-*β* ↑ and TNF-*α* ↑ beforehand and after viral inoculation to defense RSV	

Xia Sang Ju Granule (XSJG)	*Prunella vulgaris L.*	66.23%	Fruit spike	RSV replication ↓; the virus titer ↓; the XSJG group: TC50: 800 *μ*g/mL; IC50: 544.59 *μ*g/mL; TI: 1.47; the ribavirin group was similar to the XSJG group (*P* > 0.05); no adverse reaction was found	[53]
	*Dendranthema indicum*	23.18%	Flower		
	*Mulberry leaf*	10.60%	Leaf		

Th1 cell: T helper 1 cell; Th2 cell: T helper 2 cell; Th17-cell: T helper 17 cell; GATA3: transcription factors GATA binding protein 3; STAT6: signal transducer and activator of transcription 6; ROR*γ*T: retinoid-related orphan receptor gamma t; T-Bet: T box expressed in T cells; IL: interleukin; A549: human lung carcinoma cell; TC50 is 50% of toxic concentration; IC50 is the concentration of the sample required to inhibit virus-induced CPE 50%; CC50 is the concentration of the 50% cytotoxic effect; IFN-*β*: interferon *β*; IFN-*γ*: interferon-*γ*; TNF-*α*: tumor necrosis factor-*α*.

**(b):** 

Names	Composition			Mechanisms and results	References
	Plant or mineral	Weight (g)	Used part		
Jin Xin oral liquid (JOL)	*Ephedra sinica Stapf*	3	Stem	Lung inflammation ↓; RSV replication ↓; the viral load in the lung tissues ↓	[54]
	*Prunus armeniaca L.*	10	Seed		
	*Morus alba L.*	10	Velamen	During early stage infection (on days 2 and 4 after infection), the TLR3-IRF3-IFN-*β* signaling pathway ↓: IFN-*β* ↓; TLR3 ↓; IRF3 (p-IRF3) ↓; the expression of SOCS1 ↓	
	*Scutellariae baicalensis Georgi*	10	Rootstalk		
	*Peucedanum praeruptorum Dunn*	10	Root		
	*Lepidium apetalum Willd.*	10	Seed	During the later stage of infection (7 d after infection), the TLR3-IRF3-IFN-*β* signaling pathway ↑: IFN-*β* ↑; TLR3 ↑; IRF3 (p-IRF3) ↑; the expression of SOCS1 ↓	
	*Polygonum cuspidatum Sieb.*	10	Root		
	*Gypsum (CaSO*_4_ ·2*H*_2_ *O*)	15	—		

Qing Fei oral liquid (QOL)	*Ephedra sinica Stapf*	4	Stem	A multicentered single-blind, random controlled clinical study of 166 children in Nanjing in China showed that the curative and the effective rate of the QOL group: 80.00%; 19.38%; the ribavirin group: 41.14%; 41.14%; no adverse reaction was found	[55]
	*Prunus armeniaca L.*	10	Seed		
	*Morus alba L.*	10	Velamen		
	*Bombyx mori Linnaeus*	6	Bombycidae		
	*Peucedanum praeruptorum Dunn*	10	Root		
	*Lepidium apetalum Willd.*	6	Seed	Airway inflammation ↓; IL-6 ↓; IL-8 ↓	[56]
	*Polygonum cuspidatum Sieb.*	12	Root		
	*Gypsum (CaSO*_4_ ·2*H*_2_ *O*)	24	—	RSV replication ↓; regulating immunabitity INF-*γ* ↑	[57]
	*Salvia miltiorrhiza Bge*	6	Rootstalk		
	*Polygonum bistorta L.*	12	Rootstalk		

TLR3: toll-like receptors 3; IRF3: interferon regulatory factor 3; IFN-*β*: interferon *β*; SOCS1: suppressor of cytokine signaling 1; IL-6: interleukin-6; IL-8: interleukin-8; IFN-*γ*: interferon-*γ*; A549: human lung carcinoma cell; TC50 is 50% of toxic concentration; IC50 is the concentration of the sample required to inhibit virus-induced CPE 50%; therapeutic index (TI): TC50/IC50.

**(a):** 

Names	Composition			Mechanisms and results	References
	Plant or mineral	Weight (g)	Used part		
Modified Ding Chuan Tang (MDD)	*Salviae miltiorrhizae Radix*	12	Root	MDD exhibited significant antiviral and anti-inflammatory effects: lung viral loads ↓; the MDD group: IC50: 27.2 *μ*g/mL; CC50: >1000 *μ*g/mL; SI: >36.8; the ribavirin group: IC50: 2.8 *μ*g/mL; CC50: 41.6 *μ*g/mL; SI: 14.9 eotaxin ↓; IL-4 ↓; IFN-*γ* ↓ in serum and lung tissue; TLR4 ↓; NF-*κ*B ↓ in the lung tissue	[67]
	*Scutellariae Radix*	9	Root		
	*Farfarae Flos*	10	Flower		
	*Ephedra sinica Stapf*	4	Stem		

Ge Gen Tang (GGT)	*Cinnamomum cassia Blume*	3.0	Twig	GGT was more effective given before than after viral inoculation (*P* < 0.0001); viral attachment ↓; viral internalization ↓; in A549 cells: IC50 was 8.8 *μ*g/mL (2 h before); IC50 was 27.1 *μ*g/mL (1 h before); IC50 was 184.7 *μ*g/mL (1 h after); the immune-modulatory effect: IFN-*β* ↑; GGT will not increase TNF-*α* secretion, but rather inhibit TNF-*α* secretion in HEp-2 cells	[1]
	*Ephedra sinica Stapf*	4.5	Stem		
	*Glycyrrhiza uralensis Fischer et DC*	3.0	Radix		
	*Paeonia lactiflora Pallas*	3.0	Radix		
	*Pueraria lobata Ohwi*	6.0	Radix		
	*Zingiber officinale Roscoe*	4.5	Root-like stem		
	*Ziziphus jujuba Mill.var. inermis (Bge.) Rehd.*	4.0	Fruit		

Jia Wei Yu Ping Feng Tang (JYT)	*Radix Astragali*	27.7	Root	Viral attachment ↓; RSV internalization ↓; lung lesions and complication ↓; inhibiting the expression of ICAM1; in A549 cells: the JYT group: CC50: 1373.37 ± 2.8 *μ*g/mL, IC50: 97.0 *μ*g/mL; the ribavirin group: CC50: 579.47 ± 7.4 *μ*g/mL; the IC50 value of ribavirin was similar; JYD could inhibit RSV infection in all three modes of treatment but was more effective in the simultaneous and posttreatment modes (*P* < 0.05)	[68]
	*Rhizoma Atractylodis Macrocephalae*	16.7	Rhizome		
	*Radix Saposhnikoviae*	16.7	Root		
	*Flos Lonicerae japonicae*	16.7	Flower bud		
	*Rhizoma Dryopteris Crassirhizoma*	11.1	Rhizome		
	*Pericarpium Citri Reticulatae*	11.1	Fruit		

IC50 is the concentration of the sample required to inhibit virus-induced CPE 50%; CC50 is the concentration of the 50% cytotoxic effect; selective index (SI) or therapeutic index (TI): CC50/IC50; NF-*κ*B: nuclear factor-kappa B; IL: interleukin; IFN-*γ*: interferon-*γ*; IFN-*β*: interferon *β*; TLR4 inhibitor: toll-like receptor 4; A549: human lung carcinoma cell; HEp-2: human larynx epidermoid carcinoma cell; ICAM-1: intercellular cell adhesion molecule-1.

**(b):** 

Names	Composition			Mechanisms and results	References
	Plant or mineral	Weight (g)	Used part		
Liu He Tang (LHT)	*Agastache rugosa*	2.0	Whole plant	LHT was more effective when given before viral inoculation (*P* < 0.0001); viral attachment (*P* < 0.0001) ↓; RSV internalization (*P* < 0.0001) ↓; CC50: 3000 *μ*g/mL; IC50: 34.2–82.8 *μ*g/mL; IC50 value of ribavirin was similar; LHT stimulated epithelial cells to secrete IFN-*β* and TNF-*α* to counteract HRSV infection before infection becomes established	[69]
	*Amomum villosum Lour.*	1.5	seed		
	*Atractylodes macrocephala Koidz.*	2.0	Root-like stem		
	*Chaenomeles sinensis*	2.0	Fruit		
	*Dolichos lablab L.*	2.0	Seed		
	*Glycyrrhiza uralensis Fischer* *et DC*	1.5	Radix		
	*Magnolia officinalis Rehd. et Wils.*	2.0	Bark		
	*Panax ginseng C.A. Meyer*	1.5	Root		
	*Pinellia ternata (Thunb.) Breit.*	1.5	Root and stem		
	*Poria cocos (Schw.) Wolf*	2.5	Sclerotium		
	*Prunus armeniaca L.*	1.5	Seed		
	*Zingiber officinale Roscoe*	1.5	Root-like stem		
	*Ziziphus jujuba Mill.* *Var. inermis (Bge.) Rehd.*	1.5	Fruit		

Xiao Qing Long Tang (XQLT)	*Ephedra sinica Stapf*	9.0	Stem	Hot water extract of XQLT dose-dependently inhibited HRSV-induced plaque formation when given before viral inoculation (*P* < 0.0001); viral attachment ↓ (*P* < 0.0001); RSV internalization ↓ (*P* < 0.0001); CC50: 300 *μ*g/mL; IC50: 34.2–82.8 *μ*g/mL; IC50 value of ribavirin was similar; in A549 cells: IC50 was 22.6 *μ*g/mL (2 h before); IC50 was 50.4 *μ*g/mL (1 h before); IC50 was 128.1 *μ*g/mL (1 h after); secreting IFN-*β* ↑ before and after viral inoculation to counteract viral infection (*P* < 0.0001)	[70]
	*Cinnamomum cassia Blume*	6.0	Twig		
	*Paeonia lactiflora Pall.*	9.0	Root		
	*Glycyrrhiza uralensis Fisch.*	6.0	Root		
	*Zingiber officinale Rosc.*	3.0	Rhizome		
	*Pinellia ternata (Thunb.) Breit.*	9.0	Tuber		
	*Asarum heterotropides F Maekawa*	3.0	Whole plant		
	*Schisandra chinensis (Turcz.) Baill.*	3.0	Fruit		

IC50 is the concentration of the sample required to inhibit virus-induced CPE 50%; CC50 is the concentration of the 50% cytotoxic effect; selective index (SI) or therapeutic index (TI): CC50/IC50; IFN-*β*: interferon *β*; TNF-*α*: tumor necrosis factor-*α*; A549: human lung carcinoma cell.

**Table 6:** The mechanisms of TCMHs-based prevention and treatment of RSV.

Prevention	Treatment
Viral attachment ↓ [1, 24, 29, 32, 68–70]; viral internalization ↓ [1, 24, 29, 32, 68–70]	RSV entry ↓ [24, 32–49]

IFN-*β*, TNF-*α* production → viral replication ↓[1, 24, 29, 32, 54, 69, 70]	RSV replication ↓ [32–49, 51–55, 57]

IFN-*γ* production → RSV viral infection ↓ [30, 31, 50, 57, 67]	Syncytium formation of RSV ↓ [26]

The survival of human lung epithelial cells ↑ [31]	Lung inflammation ↓ [30, 50–52, 54–56, 67, 68]

Activate the immune system [30, 50, 57]	F protein expression of RSV ↓ [50]

Blocked proinflammatory gene expression [31, 54, 68] (in the human alveolar epithelial cell line)	Viral loads in serum and lung tissue ↓ [51–54, 67]; no adverse reaction was found [53, 55]
